# 

**Published:** 1882-01

**Authors:** 


### CONTENTS.

### COMMUNICATIONS.

On Specialism; and on the Influence of Medical Science on Modern Civilization ........ 1
On the Remedies used to Promote Absorption of Inflammatory and other Morbid Products ........ 9
Writer's Cramp ........ 12

### SELECTIONS.

Our Medical Literature ........ 13
Wheat Phosphates ........ 30
The Innervation of the Salivary Glands ........ 43
A New Dental Disease ........ 44
Salicilic Acid as a Prophylactic ........ 44

### CORRESPONDENCE.

Transmission of Traumatic Lesion ........ 45
Relative Mortality Amongst White and Colored Races ........ 46

### EDITORIAL.

A New Dental College ........ 47
The Ohio State Dental College ........ 48
Sanitary ........ 49
The Medical and Dental Laws ........ 49
Mortality Statistics ........ 50

